# Association Between Endometriosis and Subsequent Risk of Sjögren’s Syndrome: A Nationwide Population-Based Cohort Study

**DOI:** 10.3389/fimmu.2022.845944

**Published:** 2022-05-03

**Authors:** Yung-Hsiang Chao, Chin-Hsiu Liu, Yu-An Pan, Fu-Shun Yen, Jeng-Yuan Chiou, James Cheng-Chung Wei

**Affiliations:** ^1^ Institute of Medicine, Chung Shan Medical University, Taichung, Taiwan; ^2^ Department of Allergy, Immunology & Rheumatology, Chung Shan Medical University Hospital, Taichung, Taiwan; ^3^ Rheumatology and Immunology Center, China Medical University Hospital and College of Medicine, China Medical University, Taichung, Taiwan; ^4^ Department of Sociomedical Science, The Mailman School of Public Health, Columbia University, New York, NY, United States; ^5^ Dr. Yen’s Clinic, Taoyuan, Taiwan; ^6^ School of Health Policy and Management, Chung Shan Medical University, Taichung, Taiwan; ^7^ Graduate Institute of Integrated Medicine, China Medical University, Taichung, Taiwan; ^8^ Department of Medical Research, Taichung Veterans General Hospital, Taichung, Taiwan

**Keywords:** Sjogren’s syndrome, autoimmune disease, endometriosis, epidemiology, cohort

## Abstract

**Objective:**

The relationship between endometriosis and the ensuing risk of Sjögren’s syndrome has remained unclear. This study aims to present epidemiological evidence for this connection.

**Methods:**

This is a retrospective cohort study of endometriosis patients (ICD-9-CM 617.0-617.9 and 621.3) and matched comparison group between 2000 and 2012 in the National Taiwan Insurance Research Database. After age matching, we analyzed the association between endometriosis and Sjögren’s syndrome (ICD-9-CM 710.2). We used the Cox proportional hazard model to examine the hazard ratio of incidental Sjögren’s syndrome. Subgroup analyses on age, comorbidities, and disease duration were also performed.

**Results:**

A total of 73,665 individuals were included in this study. We identified 14733 newly diagnosed endometriosis patients and 58,932 non-endometriosis comparison group. The adjusted hazard ratio (HR) for incidental Sjögren’s syndrome was 1.45 (95% confidence interval CI=1.27-1.65) in the endometriosis group, compared to the non-endometriosis comparison group. In subgroup analysis, the adjusted HR was 1.53 (95% CI=1.25-1.88) in the age group of 20-39 and 1.41 (95% CI =1.18-1.68) in the age of 40-64. Time-vary analysis showed that endometriosis who have a follow-up time of fewer than five years (adjusted HR=1.57, 95% CI=1.32-1.87) have a significantly highest risk of having subsequent Sjögren’s syndrome.

**Conclusion:**

This population-based cohort study indicated that having a history of endometriosis puts patients at an increased risk of getting Sjögren’s syndrome afterward, especially in the age group of 20-39 and within the first five years after the diagnosis of endometriosis. Clinicians should recognize this possible association in managing endometriosis or Sjögren’s syndrome patients.

## Introduction

Endometriosis is a common estrogen-dependent gynecologic disorder. Individuals with endometriosis have a high rate of infertility. As an inflammatory disease that depends on estrogen, endometriosis impacts about 5-10% of women in their reproductive years (1) and prevalence about 20-30% in infertile women (2). According to the report of Eisenberg (3), the prevalence of diagnosed endometriosis was 10% of premenopausal women worldwide. Endometriosis is the endometrium present outside the ovaries and the pelvic peritoneum. Approximately 8.9% of the women have endometriosis in Taiwan (4). Nevertheless, little is known regarding the pathophysiology of endometriosis. Endometriosis has been reported to be associated with immune dysfunctions, such as the evasion of endometrial tissues from natural killer (NK) cell-mediated clearance, inflammation in the peritoneal microenvironment, and upregulation of interleukin (IL)-6, IL-8, and tumor necrosis factor (TNF)-α, which promote the proliferation of endometrial cells and neovascularization (5–7). Although the immune system appears to play a role in the pathogenesis of endometriosis, the association between endometriosis and autoimmune diseases is still being argued with little and conflicting evidence (8). It is not clear whether autoimmune diseases are a risk factor of endometriosis or whether these two types of diseases share similar pathological mediators. It has been indicated that autoimmune disorders could occur more often among those with endometriosis. However, endometriosis’s actual mechanism and genetic etiology are unknown (9).

As a chronic systemic autoimmune disease, Sjögren’s syndrome is characterized by xerostomia (dry mouth), keratoconjunctivitis sicca (eye dryness), and other systemic conditions (10). Sjögren’s syndrome is classified as primary Sjögren’s syndrome or second Sjögren’s syndrome. Primary Sjögren’s syndrome occurs in the absence of another rheumatic disorder. On the other hand, secondary Sjögren’s syndrome is mainly associated with autoimmune diseases such as rheumatoid arthritis, ankylosing spondylitis, systemic lupus erythematosus, and Graves’ disease (11). The hallmark of primary Sjögren’s syndrome is lymphocytic infiltration of the lacrimal and salivary glands (12). The prevalence of primary Sjögren’s syndrome is around 0.05% to 4.8% of the world population, with a 9:1 female to male ratio and is highest in Asian women (13, 14). Taiwan’s incidence of Sjögren’s syndrome was 4.98 to 8.86 of 100,000 per year (15). A complicated mechanism moderates the pathogenesis of Sjögren’s syndrome: the infiltration of lymphocytes (mainly T and B cells) in target organs within the dysregulated adaptive immunity system (16). Sjögren’s syndrome characteristics include an exacerbated T cell infiltration in exocrine glands, which is related to its inflammatory feature and disease progression (17, 18). Previous studies have revealed that autoimmune diseases such as Sjögren’s syndrome, systemic lupus erythematosus (SLE), rheumatoid arthritis (RA), and inflammatory bowel disease were more prevalent in patients with endometriosis (19). Those with endometriosis are also more at risk of having rheumatoid arthritis, according to a retrospective cohort study (20). In a systematic review of 26 case-control cohort and cross-sectional studies, endometriosis was proposed to be related to autoimmune diseases, including Sjögren’s syndrome (21). In contrast, epidemiological research explored that women with endometriosis do not have an increased prevalence of Sjögren’s syndrome (22). In addition, a nationwide cohort-based study does not provide evidence to back up prior claims of endometriosis patients being significantly more likely of getting Sjögren’s syndrome (23). Although conflicting evidence demonstrates relationships between women with endometriosis and autoimmune diseases, it requires more clinical supporting evidence for their relevance. Therefore, we evaluated the prevalence and risk of Sjögren’s syndrome in a nationwide Taiwan cohort among endometriosis patients.

## Methods

### Data Source

This study was a retrospective, population-based cohort study. We obtained the data from the National Health Insurance Research Database (NHIRD), which began in 1995 and covered more than 99% of Taiwanese. We constructed the 2000 Longitudinal Health Insurance Database (LHIRD), established by NHIRD. NHIRD has been used for various studies and collects specific information on diagnosed hospitalizations and prescriptions. Data regarding the medical services that the programmer offers are obtained by the National Health Insurance Administration and entered into the NHIRD. The database contains population-level demographic characteristics, admissions, prescription drugs after discharge, surgical procedures, and diagnostic codes. *International Classification of Diseases Ninth Revision, Clinical Modification* (ICD-9-CM) is listed to include a maximum of five diagnoses. The Chung Shan Medical University Ethics Review Board approved this study by CS15134.

### Sample Participants

We identified 20 years and older patients newly diagnosed with endometriosis during the 1 January 2000 and 31 December 2012 time period as the endometriosis population. Endometriosis was defined based on specific admission codes (ICD-9-CM 617.0-617.9 and 621.3). We used the index date as the diagnosis date of endometriosis. The comparison group was comprised of individuals without a diagnosis of endometriosis. The non-endometriosis group was matched by a 1:4 age ratio and the index date every five years for incidence density sampling. From 2000-2012, we identified 14,733 newly diagnosed endometriosis patients and 58,932 cases without endometriosis. Both groups were confirmed from the index date to the date of Sjögren’s syndrome diagnosis, no longer being involved in the NHI program or the date of the first occurrence at the end of 2013.

### Outcome, Comorbidities, and Exposure Measurement

People were being traced until death or till the study period ended (31 December 2013). The primary outcome was the incidental Sjögren’s syndrome (outpatient and inpatient setting coded as ICD-9-CM code 710.2) made by rheumatology according to the European classification criteria (14). The outcome variable in our study was Sjögren’s syndrome (24). We indicated any incidence of Sjögren’s syndrome, at least one inpatient claims data or at least two outpatients’ diagnoses within 2 years between 2000 and 2013 as a new diagnosis. We defined the index date as the first diagnosis day (25). Since the risk of producing the outcome may be affected by comorbidities, histories of diabetes mellitus (ICD-9-CM code 250), hyperlipidemia (ICD-9-CM code 272.0), coronary artery disease (ICD-9-CM 410-413, 414.01- 414.05, 414.8-414.9), congestive heart failure (ICD-9-CM 428, 397.91, 402.x1), obesity (ICD-9-CM 278, A183), stroke (ICD-9-CM 430-438), and chronic kidney disease (ICD-9-CM 403, 404, 585 and 586) were included as covariates. Meanwhile, demographic factors, including age, urbanization level, and monthly income, were adjusted in this study. The participant status determined those comorbidities before the index date.

### Statistical Analysis

We compared endometriosis and matched the comparison cohort by Student’s t-test and Chi-square test based on the differences in baseline characteristics and comorbid medical disorder. The incidence density rates (per 1,000 person-years) were analyzed to estimate incidence in both the endometriosis group and matched the comparison cohort by stratification age grouped and comorbidities grouped. We used the number of newly diagnosed Sjögren’s syndrome cases by the number of person-years at risk of Sjögren’s syndrome from 2000 to 2013 to calculate the annual incidence density rate. Also, we used the Cox proportional hazard regression models to compare Sjögren’s syndrome risk between endometriosis persons and matched comparison cohort. We analyzed the regression of the impact of variation for diabetes mellitus, hyperlipidemia, congestive heart failure, coronary artery disease, obesity, stroke, and chronic kidney disease in endometriosis cases. The significance threshold was set at α=0.05 for prior hypotheses. We used the SAS statistical software Version 9.4 to perform all statistical analyses.

## Results

A total of 73,665 individuals were included in this study. 14,733 were in the endometriosis group, and the remaining 58,932 people were in the comparison group. As a result of a successful incidence density sampling program, no significant difference was found between patients with endometriosis and the matched comparison cohort in the distributions of observation age groups, according to **Table 1**. The mean ages (SD) of the study subjects were 38.9 ( ± 10.3) and 38.9 ( ± 10.4) years old among patients with endometriosis and matched comparison cohort (Student’s t-test, *p >*0.99). Among the subjects, patients between 20 and 39 years old were dominant. The mean follow-up (SD) time is 7.27 (0.03) years in patients with endometriosis and 7.25 (0.01) years in the matched comparison cohort.

**Table 1:** Baseline characteristics of patients.

	Endometriosis (n = 14733)		Comparison group (n = 58932)		p-value*
	n	%	n	%	
**Age, years**					
20-39	7945	53.9	7945	53.9	
40-64	6537	44.4	6537	44.4	
≥65	251	1.70	251	1.70	
Mean (SD)^†^	38.9 (10.3)		38.9 (10.4)		>0.99
**Comorbidity**					
DM	292	1.98	1136	1.93	0.66
Hypertension	1646	11.1	5832	9.90	<0.01
Hyperlipidemia	1425	9.67	4456	7.56	<0.01
Coronary artery disease	658	4.47	1879	3.19	<0.01
Congestive heart failure	168	1.14	572	0.97	0.06
Obesity	226	1.53	600	1.02	<0.01
Stroke	590	4.00	1811	3.07	<0.01
Chronic kidney disease	102	0.69	338	0.57	0.09
**Urbanization level**					<0.01
1 (highest)	5374	36.5	19548	33.2	
2	4432	30.1	17851	30.3	
3	2275	15.5	10022	17.0	
4 (low)	2646	17.9	11469	19.5	
**Monthly income (NTD $)**					<0.01
<15000	6061	41.1	25146	42.7	
15000-29999	6260	42.5	26365	44.7	
≥30000	2412	16.4	7421	12.6	
**Follow-up time, year** ^†^	7.27 (0.03)		7.25 (0.01)		

*P-value using chi-square for the comparisons between with and without endometriosis.

^†^Average age and follow-up time using student’s t test for verification.

The urbanization level was categorized by the population density of the residential area into 4 levels, with level 1 as the most urbanized and level 4 as the least urbanized.

However, several demographic data(comorbidities) were significantly more frequent in patients with endometriosis than in matched comparison cohort; these included hypertension (11.1%), hyperlipidemia (9.67%), coronary artery disease (4.47%), obesity (1.53%), and stroke (4%). Urbanization level was defined as the categorization of residential areas’ population density: level 1 is the most urbanized whereas level 4 is the least urbanized. Urbanization levels and the amount of monthly income were also found to be significantly associated with endometriosis (p<0.01).

Sjögren’s syndrome incidence density rate was higher in endometriosis patients than those in the matched comparison cohort group (2.84 and 1.93 per 1,000 person-years), as shown in **Table 2**. The results revealed that patients with endometriosis had 1.45 times the risk (95% CI=1.27-1.65) of developing Sjögren’s syndrome compared to individuals without endometriosis. Advanced age between 40 and 64 years, over 65 years, hyperlipidemia, and monthly income of more than 30,000 NT dollars showed a significantly positive association with Sjögren’s syndrome. Compared with patients of urbanization 1, patients of urbanization 2,3 and 4 had an adjusted HR of 0.96 (95%CI=0.83-1.11),0.97 and 1.03, respectively, but the difference did not show significance. The risk of Sjögren’s syndrome was 1.60-fold and 2.33-fold more significant in patients of the age of 40-64 and more than 65 years old compared to that in patients of less than 40 years old (95% CI=1.40-1.82 and 95% CI=1.59-3.43, *p*<0.001), respectively. Among various types of comorbidities, the significantly elevated risk appeared in those with hyperlipidemia (adjusted HR=1.25, 95% CI=1.01-1.54) and those with a monthly income of more than 30,000 NT dollars (adjusted HR=1.19, 95% CI=1.00-1.42). However, the association between comorbidities such as diabetes mellitus, hypertension, coronary artery disease, congestive heart failure, obesity, stroke, chronic kidney disease, and urbanization level was statistically insignificant.

**Table 2:** Cox regression analyses of Sjögren’s syndrome in patients with endometriosis.

Variable	Sjögren’s syndrome			Crude HR (95%CI)	Adjusted HR† (95%CI)
	Event	PY	IR		
**Endometriosis**					
No	826	427775	1.93	1	1
Yes	305	107253	2.84	1.47 (1.29-1.68)***	1.45 (1.27-1.65)***
**Age**					
20-39	472	299915	1.57	1	1
40-64	625	227692	2.74	1.75 (1.55-1.97)***	1.60 (1.40-1.82)***
≥65	34	7421	4.58	2.94 (2.08-4.17)***	2.33 (1.59-3.43)***
**Comorbidity**					
DM					
No	1097	525171	2.08	1	1
Yes	34	9857	3.44	1.65 (1.17-2.32)**	0.98 (0.68-1.42)
Hypertension					
No	975	484528	2.01	1	1
Yes	156	50500	3.08	1.54 (1.30-1.82)***	0.93 (0.76-1.15)
Hyperlipidemia					
No	1007	499624	2.01	1	1
Yes	124	35404	3.50	1.76 (1.46-2.12)***	1.25 (1.01-1.54)*
Coronary artery disease					
No	1072	519358	2.06	1	1
Yes	59	15670	3.76	1.84 (1.41-2.39)***	1.15 (0.86-1.54)
Congestive heart failure					
No	1118	530771	2.10	1	1
Yes	13	4257	3.05	1.46 (0.84-2.53)	0.78 (0.44-1.40)
Obesity					
No	1120	530236	2.11	1	1
Yes	11	4792	2.29	1.10 (0.60-1.99)	0.96 (0.52-1.74)
Stroke					
No	1071	518464	2.06	1	1
Yes	60	16564	3.62	1.75 (1.35-2.28)***	1.20 (0.91-1.59)
Chronic kidney disease					
No	1121	532532	2.10	1	1
Yes	10	2496	4.00	1.92 (1.03-3.59)*	1.34 (0.71-2.53)
**Urbanization level**					
1 (highest)	399	182558	2.18	1	1
2	333	162214	2.05	0.94 (0.81-1.08)	0.96 (0.83-1.11)
3	178	88846	2.00	0.91 (0.76-1.09)	0.97 (0.82-1.16)
4 (low)	221	101410	2.08	0.99 (0.84-1.17)	1.03 (0.88-1.22)
**Monthly income (NTD $)**					
<15000	361	207104	1.74	1	1
15000-29999	562	250074	2.24	1.28 (1.12-1.46)***	1.04 (0.90-1.19)
≥30000	208	77850	2.67	1.52 (1.28-1.80)***	1.19 (1.00-1.42)*

PY, person-years; IR, incidence rate, per 10,000 person-years; HR, hazard ratio; CI, confidence interval.

^†^HR adjusted for age, diabetes mellitus, hyperlipidemia, coronary artery disease, congestive heart failure, obesity, stroke and chronic kidney disease.

*p<0.05, **p<0.01, ***p<0.001.

**Supplementary Table 1**. shows the results of Cox regression analyses of primary and secondary Sjögren’s syndrome in patients with endometriosis. Among the 305 endometriosis patients, there were 56 (18.36%) patients with primary Sjögren’s syndrome and 249 (81.64%) patients with secondary Sjögren’s syndrome. The incidence density rate of primary Sjögren’s syndrome was higher than secondary Sjögren’s syndrome (2.84 and 2.32 per 1,000 person-years, respectively). The risk of primary Sjögren’s syndrome was 1.40-fold (95%CI=1.21-1.62, p<0.001) in patients with endometriosis, whereas those with secondary Sjögren’s syndrome had an adjusted HR of 1.73 (95%CI=1.26-2.38, p<0.001).

**Table 3** shows the results of Cox regression stratified by age and various comorbidities. The study results revealed that among the 1,131 patients with endometriosis, 305 of them developed subsequent Sjögren’s syndrome. After adjusting for age and multiple comorbidities, patients with endometriosis were at a significantly higher risk of getting Sjögren’s syndrome than matched cohorts in the age of 20-39 years old (adjusted HR=1.53, 95% CI=1.25-1.88), in the age of 40-64 years old (adjusted HR=1.41, 95% CI=1.18-1.68), in diabetes mellitus (adjusted

**Table 3:** Cox regression analyses of Sjögren’s syndrome in patients with endometriosis stratified by age and comorbidity.

	Endometriosis						Crude HR (95%CI)	Adjusted HR† (95%CI)
	No			Yes				
	Event	PY	IR	Event	PY	IR		
**Age**		**427775**			**107253**			
20-39	339	239525	1.42	133	60390	2.2	1.55 (1.27-1.90)***	1.53 (1.25-1.88)***
40-64	459	182291	2.52	166	45400	3.66	1.45 (1.21-1.73)***	1.41 (1.18-1.68)***
≥65	28	5959	4.7	6	1463	4.1	0.87 (0.36-2.11)	0.97 (0.39-2.40)
**Comorbidity**								
DM								
No	806	419915	1.92	291	105256	2.76	1.44 (1.26-1.64)***	1.41 (1.23-1.61)***
Yes	20	7860	2.54	14	1997	7.01	2.71 (1.37-5.38)**	2.54 (1.26-5.09)**
Hyperlipidemia								
No	716	388250	1.84	259	96278	2.69	1.45 (1.26-1.68)***	1.42 (1.23-1.64)***
Yes	110	39525	2.78	46	10975	4.19	1.50 (1.06-2.12)*	1.54 (1.09-2.18)*
Coronary artery disease								
No	782	416271	1.88	290	103086	2.81	1.49 (1.30-1.71)***	1.48 (1.29-1.69)***
Yes	44	11504	3.82	15	4167	3.6	0.94 (0.52-1.69)	0.91 (0.50-1.65)
Congestive heart failure								
No	817	424512	1.92	301	106260	2.83	1.47 (1.29-1.68)***	1.44 (1.26-1.65)***
Yes	9	3263	2.76	4	993	4.03	1.43 (0.44-4.67)	1.44 (0.44-4.76)
Obesity								
No	818	424251	1.93	302	105985	2.85	1.47 (1.29-1.68)***	1.45 (1.27-1.65)***
Yes	8	3524	2.27	3	1268	2.37	1.01 (0.27-3.83)	1.19 (0.30-4.67)
Stroke								
No	784	415424	1.89	287	103040	2.79	1.47 (1.29-1.69)***	1.45 (1.27-1.66)***
Yes	42	12351	3.4	18	4213	4.27	1.27 (0.73-2.21)	1.26 (0.72-2.21)
Chronic kidney disease								
No	820	425862	1.93	301	106671	2.82	1.46 (1.28-1.67)***	1.44 (1.26-1.64)***
Yes	6	1913	3.14	4	582	6.87	2.15 (0.60-7.62)	2.58 (0.65-10.2)

PY, person-years; IR, incidence rate, per 10,000 person-years; HR, hazard ratio; CI, confidence interval.

^†^HR adjusted for age, diabetes mellitus, hyperlipidemia, coronary artery disease, congestive heart failure, obesity, stroke and chronic kidney disease.

*p<0.05, **p<0.01, ***p<0.001.

HR=2.54, 95% CI=1.26-5.09), and hyperlipidemia (adjusted HR=1.54, 95% CI=1.09-2.18). Among women with coronary artery disease, endometriosis was not related to an increased risk of Sjögren’s syndrome (adjusted HR 0.91, 95% CI=0.50-1.65). Likewise, endometriosis was not associated with an increased risk of Sjögren’s syndrome among women with congestive heart failure, obesity, stroke, and chronic kidney diseases.

**Table 4** presents Cox regression analyses of Sjögren’s syndrome in patients with endometriosis stratified by follow-up time. Compared to the cohort without endometriosis, the risk of Sjögren’s syndrome among endometriosis patients with a follow-up time of fewer than 5 years (adjusted HR=1.57, 95% CI=1.32-1.87) was significantly higher.

**Table 4:** Cox regression analyses of Sjögren’s syndrome in patients with endometriosis stratified by follow-up time.

	Endometriosis						Crude HR (95%CI)	Adjusted HR† (95%CI)
	No			Yes				
	Event	PY	IR	Event	PY	IR		
**Follow-up (yrs)**								
<5	455	58858	7.73	187	14806	12.6	1.59 (1.34-1.88)***	1.57 (1.32-1.87)***
5-8	252	121143	2.08	77	30078	2.56	1.21 (0.94-1.57)	1.19 (0.92-1.55)
>9	119	247774	0.80	41	62368	0.65	1.36 (0.95-1.94)	1.35 (0.94-1.92)

PY, person-years; IR, incidence rate, per 10,000 person-years; HR, hazard ratio; CI, confidence interval.

^†^HR adjusted for age, hyperlipidemia, and monthly income.

***p<0.001.

**Figure 1:**
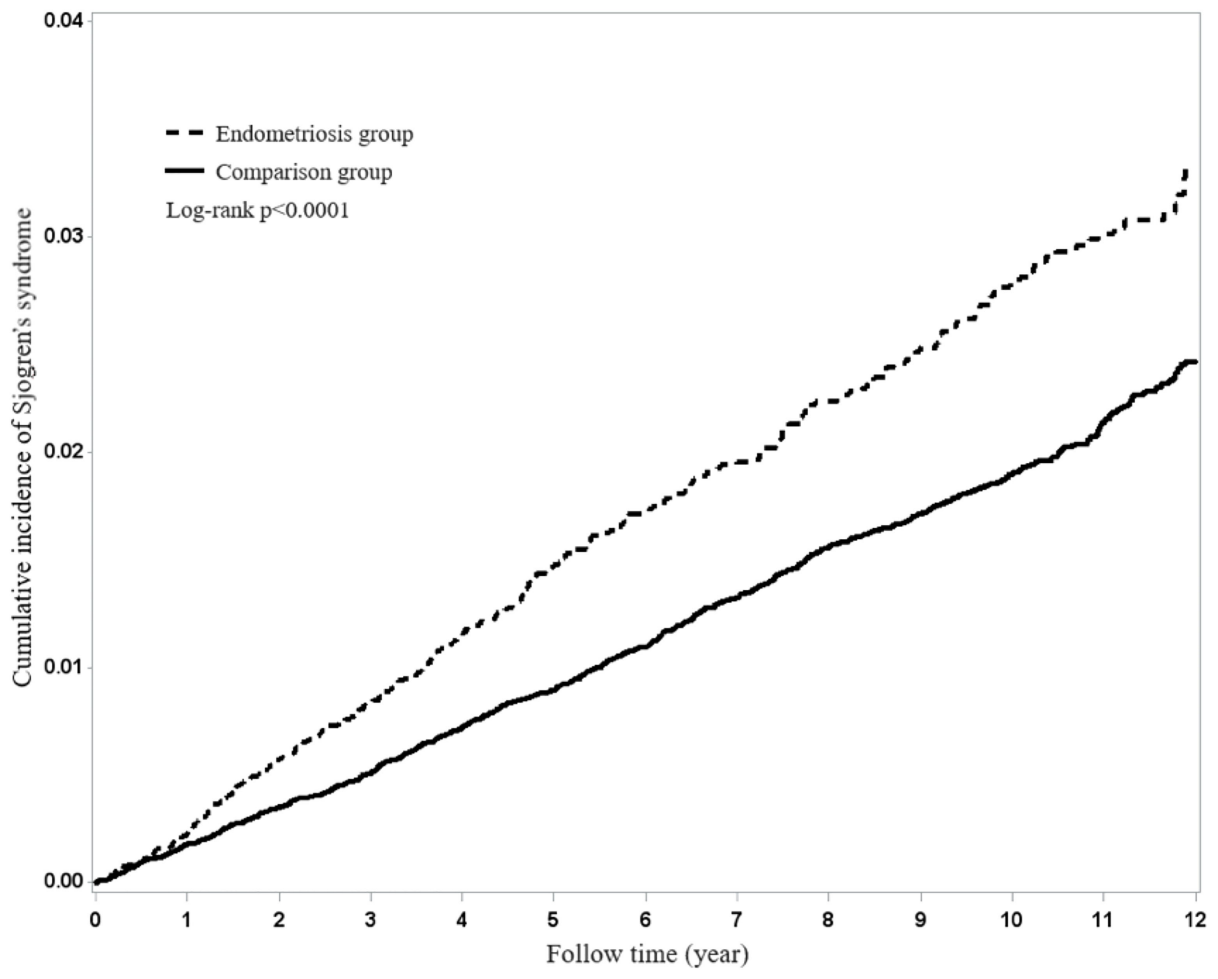
Using Kaplan-Meier survival statistics, it showed crude overall survival curves by with and without endometriosis (log-rank P<0.0001).

**Figure 1** shows that the Kaplan-Meier analysis for the cumulative incidence of Sjögren’s syndrome reveals differences between endometriosis patients and the matched comparison cohort (*p*<0.0001) as being statistically significant in Sjögren’s syndrome.

## Discussion

This study was the first retrospective cohort study that evaluated the relationship between endometriosis and the risk of developing subsequent Sjögren’s syndrome through nationwide population-based data. The results demonstrated that patients with endometriosis are associated with an increased risk of 1.45 times (95% CI=1.27-1.65) of developing Sjögren’s syndrome, as compared to those non-endometriosis comparison group. Moreover, women diagnosed with endometriosis have a higher risk of developing Sjögren’s syndrome within the first 5 years.

Our study results echoed former research and provided information analysis for patients with comorbidities by comparing cumulative incidence and Kaplan-Meier. According to a survey analysis (26), women who were diagnosed with endometriosis were found to be at a significantly (p < 0.001) higher risk of developing autoimmune diseases such as SLE, RA, and multiple sclerosis. According to new research from Fan et al. (2021), demonstrated an increased risk of SLE (adjusted HR= 2.37, 95% CI=1.35-4.14) compared to the non-endometriosis patients (27). In addition, another study suggested that women with endometriosis were significantly associated with rheumatoid arthritis (20). An immunological and inflammatory etiology has been speculated, as indicated by increased concentrations of activated macrophages, cytokines, T cells, and B cells (28). A study pointed out that endometriosis patients can be differentiated from those without by measuring their relative serum IL-6 and PF TNF-α levels (29). Many studies suggest that women with endometriosis may have a hormonal imbalance and be prone to other immune surveillance-related imbalances such as suppressed cell-mediated immunity (high T, B, and NK cell counts, but lessened activity) and increased antibody-mediated immune response (high serum levels of immunoglobulin IgA, IgG, IgM, and anti-endometrial antibodies) (30). In addition, inflammatory cytokine concentrations like IL-1, IL-6, and TNF-α were elevated among endometriosis patients (20). Patients with endometriosis also present with other chronic inflammatory diseases, chronic inflammation, and increased oxidative stress (31). As one of the most prevalent autoimmune diseases, Sjögren’s syndrome predominantly affects the lacrimal and salivary glands and thus, results in sicca symptoms (25). The possible mechanisms of endometriosis in Sjögren’s syndrome remain largely uncertain. While various other studies from different countries have examined the relationship between endometriosis and Sjögren’s syndrome risk, the studies have not yet reached a consensus. Whether Sjögren’s syndrome is a part of etiology in endometriosis or a secondary response to endometriosis deserves further genetic and biological studies for exploration.

Women aged 40-64 and women more than 65 years old have a 1.60-fold and 2.33-fold risk of having Sjögren’s syndrome compared to patients less than 40 years old, respectively. A systematic review on the epidemiology of primary Sjögren’s syndrome revealed that the mean age was 56.16 years (32). According to a 2014 population-based study in Taiwan by Yu et al, the percentage of patients with primary Sjögren’s syndrome was 73.1% and that of patients with secondary Sjögren’s syndrome was 20.3% (11). Our study cohort showed a higher percentage of secondary Sjögren’s syndrome in endometriosis patients may be association between underlying diseases, such as rheumatoid arthritis, systemic lupus erythematosus, or other autoimmune diseases. Research from Ma (33) used the Cox proportional hazard regression model analysis to show that patients with endometriosis have a significant risk of Sjögren’s syndrome (adjusted HR=1.5, 95% CI=1.27-1.77; p<0.05) than non-endometriosis. Our result is in agreement with these findings. Among various comorbidities, the risk of Sjögren’s syndrome was significantly higher only in patients with hyperlipidemia. In addition, a monthly income of more than 30,000 NT dollars was significantly associated with an increased risk of Sjögren’s syndrome. However, the association between other comorbidities, including diabetes mellitus, hypertension, coronary artery disease, congestive heart failure, obesity, stroke, and chronic kidney disease was not statistically significant. Furthermore, when the patients were stratified by comorbidities, we found that the risk of Sjögren’s syndrome was significantly increased in women with endometriosis only among those with hyperlipidemia and diabetes. In contrast, the risk of Sjögren’s syndrome was not significantly increased in women with other comorbidities, including coronary artery disease, congestive heart failure, obesity, stroke, and chronic kidney disease.

This study is noteworthy because of the large sample size from a nationwide population-based cohort and its long-term follow up for 13 years.

Nevertheless, several limitations ought to be granted attention in our study. Since the diagnosis with ICD-9 CM was confirmed by different physicians, referral bias might exist. However, we did apply a strict diagnostic definition in this study to minimize this potential problem. The other limitation is that Taiwan NHIRD does not have information on the patient’s socioeconomic status, personal health behaviors, toxic habits data, family history, and inflammatory biomarkers. Because of these confounding factors, the association between Sjögren’s syndrome and endometriosis could be biased. Since we adjusted for various comorbidities and stratified analysis and regression modeling, unmeasured confounding factors may be lessened in the results.

## Conclusion

This study demonstrated that those with a history of endometriosis increased the risk of developing subsequent Sjögren’s syndrome. Such a development was mainly observed during the first five years of patients’ endometriosis diagnosis. Future research needs to consider using the underlying biological mechanisms of these correlations. Therefore, clinicians are advised to monitor individuals with endometriosis or Sjögren’s syndrome appropriately.

## Data Availability Statement

The original contributions presented in the study are included in the article/**Supplementary Material**. Further inquiries can be directed to the corresponding author.

## Author Contributions

Y-HC drafted the manuscript. J-CW revised the manuscript critically. All authors approved the final version of the manuscript, contributed to the conception, design, and interpretation of the work substantially.

## Conflict of Interest

The authors declare that the research was conducted in the absence of any commercial or financial relationships that could be construed as a potential conflict of interest.

## Publisher’s Note

All claims expressed in this article are solely those of the authors and do not necessarily represent those of their affiliated organizations, or those of the publisher, the editors and the reviewers. Any product that may be evaluated in this article, or claim that may be made by its manufacturer, is not guaranteed or endorsed by the publisher.
