# Supplementary material for: Association Between Endometriosis and Subsequent Risk of Sjögren’s Syndrome: A Nationwide Population-Based Cohort Study
Source: Front Immunol. 2022 May 3;13:845944. doi: 10.3389/fimmu.2022.845944 (PMC9110644; doi:10.3389/fimmu.2022.845944)
Supplement: Supplementary file 1 [file Table_1.pdf]

Supplemental Table 1. Cox regression analyses of primary *Sjögren syndrome* & secondary *Sjögren syndrome* in patients with endometriosis.

	Event	PY	IR	Crude HR (95%CI)	Adjusted HR† (95%CI)
	**Sjogren syndrome (primary)**				
**Endometriosis**					
No	124	427775	1.93	1	1
Yes	56	107253	2.84	1.42 (1.23-1.64)***	1.40 (1.21-1.62)***
	**Sjogren syndrome (secondary)**				
**Endometriosis**					
No	702	427775	1.64	1	1
Yes	249	107253	2.32	1.81 (1.32-2.48)***	1.73 (1.26-2.38)***

PY, person-years; IR, incidence rate, per 10,000 person-years; HR, hazard ratio; CI, confidence interval;

†HR adjusted for age, diabetes mellitus, hyperlipidemia, coronary artery disease, congestive heart failure, obesity, stroke and chronic kidney disease.

***p<0.001
